# Deep Learning Approach for the Localization and Analysis of Surface Plasmon Scattering

**DOI:** 10.3390/s23198100

**Published:** 2023-09-27

**Authors:** Jongha Lee, Gwiyeong Moon, Sukhyeon Ka, Kar-Ann Toh, Donghyun Kim

**Affiliations:** School of Electrical and Electronic Engineering, Yonsei University, Seoul 03722, Republic of Korea; jongha24@yonsei.ac.kr (J.L.); ansrnldud@yonsei.ac.kr (G.M.); s.h.ka@yonsei.ac.kr (S.K.); katoh@yonsei.ac.kr (K.-A.T.)

**Keywords:** surface plasmon resonance, neural network, reconstruction, microscopy, Y-Net

## Abstract

Surface plasmon resonance microscopy (SPRM) combines the principles of traditional microscopy with the versatility of surface plasmons to develop label-free imaging methods. This paper describes a proof-of-principles approach based on deep learning that utilized the Y-Net convolutional neural network model to improve the detection and analysis methodology of SPRM. A machine-learning based image analysis technique was used to provide a method for the one-shot analysis of SPRM images to estimate scattering parameters such as the scatterer location. The method was assessed by applying the approach to SPRM images and reconstructing an image from the network output for comparison with the original image. The results showed that deep learning can localize scatterers and predict other variables of scattering objects with high accuracy in a noisy environment. The results also confirmed that with a larger field of view, deep learning can be used to improve traditional SPRM such that it localizes and produces scatterer characteristics in one shot, considerably increasing the detection capabilities of SPRM.

## 1. Introduction

Advanced imaging techniques are crucial in various fields, such as science, medicine, and technology. These techniques enable the visualization and understanding of different objects and systems at various scales. For instance, microscopic techniques enable the observation of microscopic events that are invisible to the naked eye. Label-free microscopic imaging has become increasingly important as it bypasses the complex labeling process, eliminating the need to consider interactions between the label and the sample [1,2,3,4,5].

Among the many label-free techniques are those that rely on surface plasmon resonance (SPR), in which the surface plasmon (SP) represents the collective oscillation of electrons caused by momentum matching with incident electromagnetic waves on a boundary, most often a dielectric–metal interface [6,7,8,9]. Under specific conditions, these oscillations create an evanescent wave that extends to the surrounding medium. The wave is highly sensitive to variations on the sample surface, which creates possibilities for the label-free detection and imaging of objects near the surface.

SPR microscopy (SPRM) combines the characteristics of SPR and microscopy to introduce a label-free detection method with high sensitivity and throughput by acquiring the refractive index distribution of an object. Advancements in nanofabrication and optical analysis techniques have further improved the possibilities and capabilities of this method for imaging and detecting smaller signals and interactions [10,11,12,13,14,15,16,17,18].

In this study, we investigated interferometric plasmonic microscopy (iPM), a modified version of SPRM, with improved sensitivity and spatial resolution. The optimization of detection methods and image-processing techniques in iPM has enabled the detection of single exosomes with miniscule signals [19]. Although iPM may detect small objects such as nanoparticles, information extraction using traditional methods remains limited due to the complex information extraction process, which we explored using deep learning.

Deep learning has attracted significant interest over the past decade because of its ability to learn features and utilize them in image analysis. Since the success of AlexNet in the 2012 ImageNet competition demonstrated the promise of deep convolutional neural networks (CNNs) in the area of image analysis, CNNs have been researched intensively for possible applications including plasmonics [20,21,22,23,24,25,26,27,28,29,30,31]. In this study, we explored a deep learning network to analyze and extract data such as scatterer location, amplitude, and phase from iPM images. We aimed to utilize the feature extraction properties of CNNs to image nanoparticles as objects in a complex noisy environment dominated by SP scattering. We also extracted other scattering parameters to simulate the iPM and compared the reconstructed image with real-world images to confirm the validity of our method. In contrast to previous studies that used deep learning in a limited manner for the classification task in SPRM, e.g., to determine the number of scatterers [32], identify extracellular vesicles [33], and predict the phase response of an image [34], this study used a trained network to effectively localize an object and extract spatial and feature information regarding scattering objects in a noisy environment from one-shot image acquisition. Label-free molecular detection assays can directly benefit from a deep learning approach, which provides considerably improved detection capabilities in plasmon-based imaging and microscopy techniques.

## 2. Materials and Methods

### 2.1. Scattering Simulation Model

**Figure 1:**
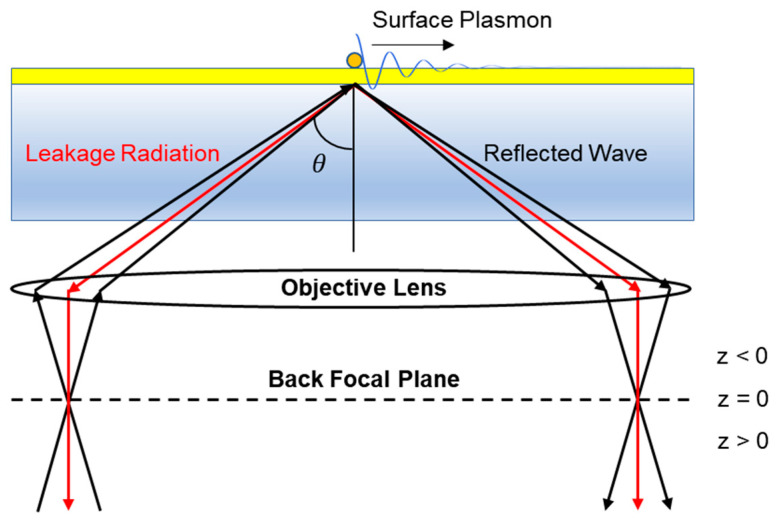
Schematic of iPM based on the superposition of the reflected wave (black) and leakage radiation (red) associated with the scattering of surface plasmons. The combined radiation creates interferometric patterns at the imaging plane. The arrows represent beam rays (z: displacement of the back focal plane; θ: angle of light incidence).

As a technique that enhances the spatial resolution and sensitivity of traditional SPRM for detecting single nanoparticles such as exosomes, the principle of iPM can be summarized as common-path interferometry [35]. The scattering model of iPM in the Kretschmann configuration for a nanostructured object is illustrated in Figure 1. Because SPR occurs when the incident light is transverse magnetic (TM), the incident wave is assumed to be TM polarized at λ = 633 nm. SPs may be scattered by an object and localized with a significantly amplified light field for various imaging and sensing applications [36,37,38,39,40].

The collected light can be modeled as a superposition of the reflected wave from the dielectric–metal interface and the object wave created by scattering caused by nanoparticles on the sample surface. The reflected wave can then be modeled as a plane wave, as shown in Equation (1):

(1) $$E_{R}\left(x,y\right)=e^{-ik\left(ycos\theta +xsin\theta \right)}$$

The reflected wave is a plane wave propagating in the dielectric region, and *k* (=$k^{\prime }+{jk}^{\prime \prime }$) is the complex wavenumber of the dielectric material. θ is the incident angle of the electromagnetic wave with respect to the normal to the interface. The variables *x* and *y* represent the coordinates of the plane normal to the sample surface aligned with the direction of electromagnetic wave propagation. Moreover, the object wave can be modeled as a spherical wave with a scattering particle located at the center, as shown in Equation (2).

(2) $$E_{O}\left(r,\;r^{\prime },z\right)=\alpha E_{R}\left(r^{\prime }\right)\cdot e^{-2k^{\prime \prime }\left|r-r^{\prime }\right|}e^{-ik^{\prime }\left|r-r^{\prime }\right|+\sqrt{{\left|nk_{0}\right|}^{2}-{\left|k_{sp}\right|}^{2}z}}$$

An object wave is an evanescent field that decreases from the scatterer. $r^{\prime }$ denotes the location of a nanoparticle, and *r* is the location on the sample [41]. The rate of oscillation and the exponential decrease in magnitude are determined by *k*′ and *k*″, which are determined by the complex permittivity of the materials used in the experiment. The displacement of the back focal plane (*z*) is shown in Figure 1. The combination of these electromagnetic wave components creates the final intensity of the output wave, as expressed in Equation (3).

(3) $$I={\left|E_{R}+E_{O}\right|}^{2}={\left|E_{R}\right|}^{2}+{\left|E_{O}\right|}^{2}+E_{R}E_{O}^{*}+E_{R}^{*}E_{O}$$

An iPM image is obtained by acquiring the background image and subtracting it from the object image. An object wave has a much smaller amplitude than the reflected wave; therefore, the amplitude of the object wave can be ignored. The final intensity of the iPM image can then be expressed using Equation (4).

(4) $$I^{\prime }={\left|E_{R}+E_{O}\right|}^{2}-{\left|E_{R}\right|}^{2}\approx E_{R}E_{O}^{*}+E_{R}^{*}\;E_{O}$$

The iPM image is highly dependent on the object wave, enabling the increased sensitivity of the scatterer and thus improving the ability of SPRM [42].

The final iPM image depends on the other parameters of the optical configuration. Changing the focus by moving the back focal plane can dramatically modify the received image. Other factors such as film thickness, propagation angle, incident angle, and scattering particle characteristics can also affect an image.

### 2.2. Optical Configuration

**Figure 2:**
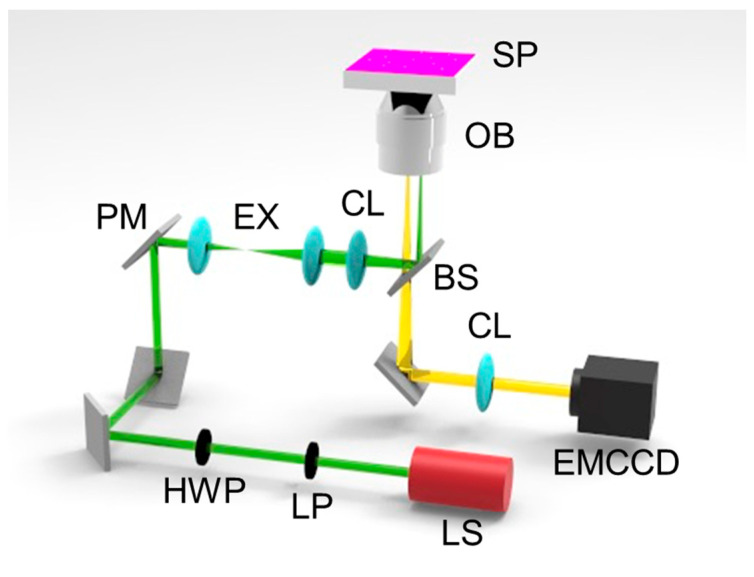
Schematic of the optical configuration used to obtain iPM images (SP: sample plane consisting of gold film and nanostructures, OB: objective lens, BS: beam splitter, CL: converging lens, EX: beam expander, PM: piezoelectric mirror, HWP: half-wave plate, LP: linear polarizer, LS: light source, and EMCCD: camera detector). The combination of HWP and LP with LS produces TM incident light, which is used to excite SPR and obtain SPRM images.

The optical configuration used to acquire the iPM images is shown in Figure 2. The Kretschmann configuration was used, and an inverted microscope (Eclipse Ti-U, Nikon, Tokyo, Japan) was used to obtain images. A continuous-wave HeNe laser (25-lhp-925-230, Melles Griot, Carlsbad, CA, USA) radiating 633 nm electromagnetic waves was employed as the light source. The laser was polarized using a linear polarizer and a half-wave plate to produce a TM-polarized electromagnetic wave. The laser was modulated using lenses such that it irradiated the entire field of view of the camera. A piezoelectric mirror (S-334.2SL, Physik Instrumente, Karlsruhe, Germany) was used to change the incident angle of the light to induce SPR. An output image was captured by an objective lens with a numerical aperture of 1.49 (CFI Apo TIRF 100XC Oil, Nikon) before being separated from the incident light through a beam splitter and acquired using an electron-multiplying charge-coupled device (EMCCD) camera (1024 × 1024 pixels, 13 μm pixel size, iXon Ultra 888, Andor, Belfast, UK) as an imaging detector. After optical magnification, the size of a single pixel was approximately 93 nm.

The spatial difference method was used to capture iPM images. An image without an object was taken as the background. The field of view was changed to obtain an image of the area containing the nanoparticles. The difference from the background image was the final iPM image.

Two types of sample objects were used in this study: an electron beam resist-based nanopost array and a gold film with gold nanoparticles. The samples were created by first cleaning a BK7 glass slide, which was evaporated to form a 50 nm thick gold film on the glass. The electron beam resist sample was then coated with an electron beam resist (AR-N 7520, Allresist, Strausberg, Germany) at a 4000 rpm angular velocity to create a 400 nm thick coating. The electron beam resist was used to create the nanopost array using electron beam lithography for a period of 20 μm.

Gold nanoparticles were added to the second sample using a gold nanoparticle solution (#742031, Sigma-Aldrich, St. Louis, MO, USA) with particles possessing a diameter of 100 nm and an optical density of 1 suspended in a 0.1 mM PBS solution. The solution was diluted to a tenth of the original concentration and dropped onto the sample. After evaporation, only nanoparticles remained on the sample.

### 2.3. Modified Y-Net

The U-Net neural network model is a fully convolutional neural network first introduced for biomedical image segmentation and has proven to be an effective model for image-to-image tasks [43]. The U-Net architecture can be divided into contractive and expansive paths. The contractive path contains convolutional and downsampling layers that reduce the spatial information while increasing the feature information. The expansive path uses intermediate results from the contractive path and the upconvolution of the contractive path output to combine features and spatial information. This process ensures that the image-to-image network produces the output of an image that contains spatial information and is aware of the feature information, thereby increasing the image-to-image task capability.

Y-Net utilizes the feature extraction power of the contractive path of U-Net to extract patch-specific data from the input images [44]. The end of the contractive path is used to create a separate output from the traditional U-Net output while maintaining the fast training speed of a fully convolutional neural network. Patch-specific data are used to further amplify the classification ability of the traditional U-Net.

**Figure 3:**
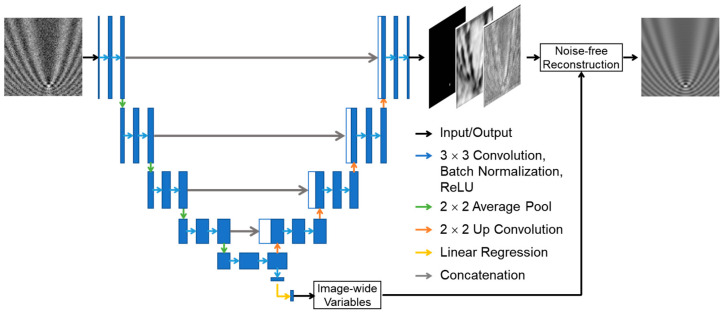
The modified Y-Net neural network model and the image reconstruction pipeline. The trained model was used to obtain image-wide variables and scatterer characteristics. The extraction of image-wide variables from the intermediate output for noise-free reconstruction through three image channels, i.e., probability map, amplitude, and phase, represents the modification to implement the Y-Net model. Legends on the right show the pipeline flow.

In this study, Y-Net was engineered to create a regression output from the end of the contractive path to obtain a significantly larger amount of information. The final model used in this study is shown in Figure 3. To implement the Y-Net model, we added a convolutional layer with an activation layer and regression layer to the U-Net at the end of the contractive path. The final intermediate output before the expansive path contained the feature information for the entire image. For iPM images, this layer contained information on image-wide variables such as the individual pixel size, back focal plane location, incident angle of an electromagnetic wave, propagation angle of an electromagnetic wave, and phase shift of the wave caused by the gold film thickness and optical path length. The feature extraction power of the contractive path could be used to obtain this information, and the use of a regression layer enabled the estimation of these variables for noise-free reconstruction.

The image output was a three-channel image with dimensions that were the same as the original image. The first channel was the probability that a particle existed at a particular location in the simulated input image. The second channel was the estimated particle amplitude, and the third channel was the estimated particle phase. These three variables determined the locations and characteristics of the scattering particles.

Four different loss functions were utilized during the training process. These loss functions were used to ensure that the model could estimate the scatterer characteristics and variables specific to the optical setup with high precision. The four loss functions $L_{i}$ (*i* = 1, 2, 3, and 4) were combined to create the final loss value, as shown in Equation (5).

(5) $$L=\alpha_{1}L_{1}+\alpha_{2}L_{2}+\alpha_{3}L_{3}+\alpha_{4}L_{4}$$

Loss weights $\alpha_{i}$ (*i* = 1, 2, 3, and 4) were used to ensure that the loss functions were balanced, because unweighted losses have intrinsically different average values, creating a final trained model that is proficient only in specific tasks. The weights for the four losses were adjusted iteratively for multiple trainings such that their average losses were similar and every training loss decreased with each training epoch. The model could use this loss function to learn the information stored in the iPM image. In Equation (5), the first loss function $L_{1}$ was used to determine the difference between the image-wide variables and the model regression output. These variables were features that affected the entire image formation process. These included the pixel size, SPR propagation angle, incident electromagnetic wave angle, back focal plane offset, gold film thickness, and reflected wave phase offset caused by the light propagation path. The mean squared error (MSE) function was used to quantify the difference between the model output and ground-truth values. Normalization was used to ensure the fair training of different image-wide variables.

The second loss function was the binary cross-entropy with logit loss function, e.g., BCEWithLogitsLoss in PyTorch, as detailed in Equation (6). The second loss function was used to determine the likelihood of a nanoparticle existing at a certain location in the image. In this study, the desired output was the localization of a pattern to a single pixel within an 80 pixel × 80 pixel image. A standard BCEWithLogitsLoss function with model weight ω = 1 converges to output a uniform probability map of nearly 0 s. ω was adjusted accordingly to ensure that the model did not converge to a uniform zero output.

(6) $$L_{2}=-[\omega \cdot y_{n}\cdot \log\sigma (x_{n})+(1-y_{n})\cdot \log\left(1-\sigma (x_{n})\right)]$$

In Equation (6), $x_{n}$, $y_{n}$, and $\sigma (x_{n})$ represent the input image, ground-truth output image that utilizes a binary particle output probability map, and model forward pass that produces the model output corresponding to input $x_{n}$, respectively.

The third and fourth loss functions were used to determine the amplitude and phase of the nanoparticle scatterer, respectively. When the output was an image, the only relevant data point was the values at the nanoparticle location. Therefore, the loss functions were variations of the MSE function, as expressed in Equation (7).

(7) $$L_{3,4}=-\frac{1}{N}\sum {\left[m_{n}\cdot \sigma (x_{n})-y_{n}\right]}^{2}$$

Here, *N* and $m_{n}$ denote the batch size and mask, respectively, representing the ground-truth probability map. The MSE value of the amplitude and phase of a nanoparticle was multiplied elementwise by the ground truth of the nanoparticle location to consider only the values at the location of the scatterer.

The four different losses were combined to obtain the final losses. The final loss was used for backpropagation. The weights of the neural network were updated accordingly. The model hyperparameters consisted of four loss weights ($\alpha_{i}$) and the model weight (ω) that appeared in BCEWithLogitsLoss (five parameters in total). These parameters were optimized by iterative training, in which the weights were adjusted until a model was obtained with decreasing epochs of all loss terms in the training dataset with good localization (no output with all 0 s). The model was trained using the adaptive moment estimation (Adam) optimizer for faster convergence with a cosine-annealing scheduler for the better estimation of the global minimum solution. Xavier initialization was used to ensure that the model converged without exploding or vanishing gradients. The best model was the last one without better results in the optimization process. The images were augmented using the Albumentations library [45] to simulate noise during the image acquisition process. Gaussian filters and Gaussian white noise were added to simulate the noise. The model was trained on a server computer with an Intel Xeon W-2295 CPU and NVIDIA RTX A4000 GPU. The model was trained using the PyTorch library for 200 epochs with a training dataset of 20,000 images and a validation set of 10,000 images. Based on the Y-Net output, we used the method for iPM simulation to reconstruct the image. The reconstruction output was compared with the model input to confirm the ability of the neural network.

## 3. Results

**Figure 4:**
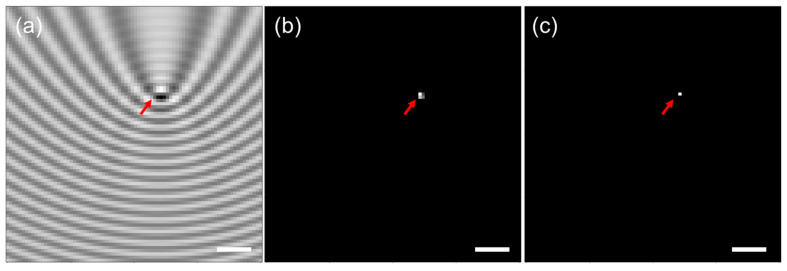
Example input and output of Y-Net. (**a**) Simulated iPM image used in the training dataset. (**b**) Probability map of the predicted location of the scatterer. (**c**) Single pixel that represents the ground truth location of the scatterer. The red arrow marks the object. Scale bar: 1 μm.

An example of the input and output of the Y-Net model for an image in the training dataset is presented in Figure 4. The interferometric pattern created through simulations was rescaled and used as the original initial input for the model, as shown in Figure 4a. The output of Y-Net was a scatterer location probability map and an image-wide variable estimation. This model could obtain the scattering parameters with high accuracy.

The final localization estimation created by the Y-Net was a probability map of the nanoparticle location, as shown in Figure 4b. A cutoff value could be used to determine the specific location of the nanoparticles in the output image. The predicted nanoparticle location for the trained model was almost identical to the ground-truth location, which is presented in Figure 4c for comparison, showing that the model could learn and predict nanoparticle locations from a given iPM image.

The neural network loss is presented in Figure S1. The four different loss functions decreased with respect to the epochs, suggesting that the model could extract features and learn the characteristics of iPM from simulated images. The decrease in the validation loss also showed that the model could predict information for images that it had not previously seen.

The model could locate the original particles with high precision, as presented in Figure 4. Note that the scatterer location loss shown in Figure S1c converged to 0 with an increasing number of epochs, indicating that the model could locate particles with extremely high accuracy, even for images in the validation set that were not used for training. The large value of *ω* used for the BCEWithLogitsLoss function ensured that even a minor offset in particle location prediction created a large loss value. The training and validation loss converging to 0 quantitatively implied that the nanoparticle localization was accurate. The model could also learn the scatterer amplitude and phase. It was suggested, however, that image-wide scattering parameters were difficult to predict. The model learned these variables with respect to the training set, which may not have translated accurately to the validation set, as shown in Figure S1 in the Supporting Information. A quantitative analysis of the localization precision was performed using 4000 images with a noise model from augmentations, suggesting that the probability of correct localization within the diffraction limit was approximately 84%, although a more realistic noise model may improve the probability (for further details, see Figure S2). In addition, a physics-aware model with more restrictions would better address problems such as overfitting.

**Figure 5:**
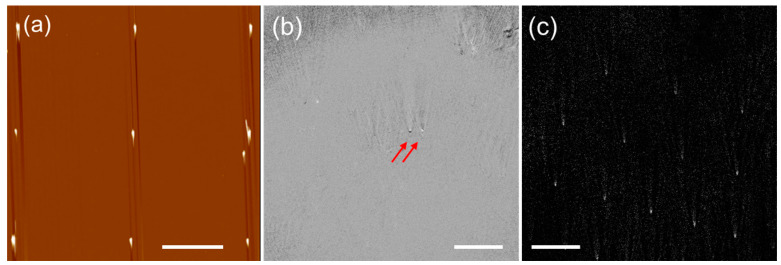
Experimentally acquired images of the nanopost array and nanoparticles. (**a**) AFM image of electron-beam-based nanopost arrays. The vertical lines are artifacts associated with high scan rates in AFM. Raw iPM image of (**b**) gold nanoparticles marked by red arrows and (**c**) nanopost array. Scale bar: 10 μm.

We now use experimental data to prove the concept of the model. Atomic force microscopy (AFM) was used to confirm the arrangement of the nanopost array, as shown in Figure 5a. The optical configuration introduced in the previous section was employed to obtain iPM images with interferometric patterns generated using gold nanoparticles and the nanopost array. The acquired images are presented in Figure 5b,c. The gold nanoparticles used to create interferometric patterns are shown in Figure 5b. Larger nanopost arrays created a much more distinguishable pattern and were more clearly observed, as shown in Figure 5c.

**Figure 6:**
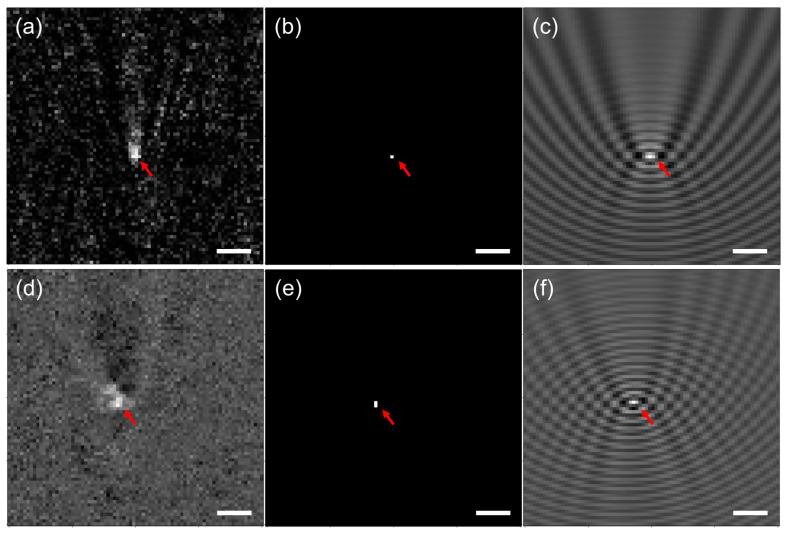
Localization and reconstruction of iPM: (**a**) experimentally acquired iPM image of a gold nanopost, (**b**) final estimated nanopost location, and (**c**) reconstruction through iPM simulation based on the probability map estimated by the Y-Net localization. (**d**–**f**) Corresponding images of a gold nanoparticle. In (**f**), the reconstruction through iPM simulation was based on the nanoparticle probability map estimated by the Y-Net localization. The red arrows mark the object location. Scale bar: 1 μm.

The interferometric pattern of a single scatterer was isolated and used as input for the modified Y-Net model. From the output of the model, an image was reconstructed using the simulation method. The Y-Net input image of a nanopost cropped in the array and the reconstructed noise-free image are shown in Figure 6a,b. In addition, the reconstruction through the iPM simulation based on the probability map estimated by Y-Net localization in Figure 6c clearly shows the interference fringe patterns of a scattering object. The same model was applied to the nanoparticles, as presented in Figure 6d–f.

The localization of the scattering particles was observed to be particularly accurate. The U-Net part of the Y-Net model was extremely effective in using features and spatial information to determine the location of particles. The local minima and maxima of the reconstructed image were similar to those of the original input image when the interference fringe observed in Figure 6a is compared with that in Figure 6c for a nanopost and that in Figure 6d with that in Figure 6f for a nanoparticle. The interferometric pattern sizes and tilts were similar in the original and reconstructed images despite weak fringe patterns in the original image.

## 4. Discussion

Although the optical resolution of iPM remained diffraction-limited, as long as the optics that captured the iPM images were unchanged, the use of deep learning based on the modified Y-Net method improved the extraction of spatial and feature information from an iPM image. This enabled the recreation of an input image with reconstruction algorithms, showing that the model was capable of extracting information and creating a noise-free reconstruction of the original image. Specifically, the network could localize an object with high accuracy, as shown by the extremely low training and validation losses for scatterer localization. The results confirmed that the method employed in this study could provide a one-shot method for scatterer localization with high accuracy and high fidelity and provided an improvement in terms of polyparametric detectability over a CNN modified from VGG19, which was applied to estimate the number of scatterers in the SPRM of light scattering [32].

For image-wide variables, deep learning using the network made it more difficult to estimate the values with high accuracy, as the validation loss decreased by a limited amount. Multiple image-wide variables could be associated with a single SPRM image, potentially resulting in an inverse problem, and could produce interfering effects on the image output among the variables that were dependent on each other. The film thickness and reference wave phase shift had almost identical effects on the interferometric pattern, enabling the model to estimate these parameters. Other image-wide variables were subject to similar scenarios in which a change in one variable had a similar effect to a change in another. Changes in pixel size and optical configuration angles could produce a similar effect, decreasing the ability of the model to predict these variables.

The reconstructed images created using the information extracted by the Y-Net model were similar to the original input image. For the training sets, the model could predict image-wide variables with high accuracy, creating a perfectly noise-free reconstructed version of the original input image. The ability of the model to analyze unknown images was reduced. The model lost accuracy because different variables had similar effects on the final output image. Whereas the model could reconstruct an image that was similar to the original input image, the fidelity of the reconstruction for images outside the training set was not guaranteed, thereby reducing the effectiveness of this approach for image analysis. Regularization may resolve this issue. In addition, a model with improved awareness of the physics of iPM is desired to further increase the model effectiveness. In this work, the scatterer characteristics and six image-wide variables were left as unknown variables for the neural network to predict. However, an appropriate physics-aware method may reduce the number of variables, creating a more accurate model with high fidelity.

The Y-Net model explored in this study used a generator to create the data. The validation on the simulated and experimental data is described in Figure S2 and Figure 6. The similarity between the experimental and generated images is obvious when Figure 6c,f are compared to Figure 4a. Nonetheless, more independent testing datasets may be crucial for the generalized performance of the model on unseen data. This method could also be extended to predict more than one object within the field of view. The simplest approach would be to divide a field of view acquired by iPM into several sub-fields for each object, as was implemented for nanoposts, and to perform the method in each sub-field (‘crop and go’). If two or more objects were close to each other, affecting each other and yet apart by more than the diffraction limit, the Y-Net model could still be applied, i.e., patterns produced by multiple particles could be used to generate validation and test datasets for deep learning. Based on these datasets, improved results were obtained using the model learned through the Y-Net architecture. If the objects were too close to each other within the diffraction limit, they could not be resolved: therefore, prediction could be difficult without additional information. Nonetheless, the measurement precision was determined by a single pixel size, which was much smaller than the diffraction limit. Image artifacts could occur due to external factors, which could affect the quality of the images or the validity of the model.

## 5. Conclusions

A CNN was demonstrated to be able to effectively determine the location and scattering parameters of an iPM image. A modified Y-Net was introduced with a regression layer at the end of the contractive path to estimate image-wide parameters. We employed simulation techniques to create a generator for the training dataset used for Y-Net. We used the generator to create a preliminary test dataset and a validation dataset to monitor the training process of Y-Net. Four loss functions were used to train the network. The trained Y-Net model was used to analyze the experimentally obtained iPM images.

The trained network could effectively localize the nanoparticle scatterer in a real-world image. The trained modified Y-Net model could extract spatial and feature information from a rescaled raw iPM image, thereby providing a simple one-shot method for analyzing images created by interferometry in a noisy environment. Reconstruction using information extracted by Y-Net, as well as a comparison with the original input image, showed that the model could estimate scattering parameters effectively. Further advancements in neural networks to cover the entire microscopic field of view while being more aware of the physics of iPM will enable a simple method for extracting all scattering parameters of iPM, significantly improving its capabilities and possibilities. Moreover, label-free imaging and detection techniques other than iPM may benefit from the deep learning approach explored in this work. In the future, we plan to confirm the effect of deep learning on images acquired using various quantitative phase imaging methods.

## Data Availability

The source code for the experiments is available from the corresponding authors upon reasonable request.

## Supplementary Materials

The following supporting information can be downloaded at: https://www.mdpi.com/article/10.3390/s23198100/s1, Figure S1: The loss values of the training set and the validation set during the training process of Y-Net for the four different types of losses; Figure S2: Some of the scattering patterns under the noise model are provided in the inset of the figure.
