# Supplementary material for: Deep Learning Approach for the Localization and Analysis of Surface Plasmon Scattering
Source: Sensors (Basel). 2023 Sep 27;23(19):8100. doi: 10.3390/s23198100 (PMC10575049; doi:10.3390/s23198100)
Supplement: Supplementary file 1 [file sensors-23-08100-s001.zip › sensors-2554421-supplementary.pdf]

Deep Learning Approach for the Localization and Analysis of Surface Plasmon Scattering

Jongha Lee, Gwiyeong Moon †, Sukhyeon Ka, Kar-Ann Toh and Donghyun Kim *

School of Electrical and Electronic Engineering, Yonsei University, Seoul 03722, Republic of Korea; jongha24@yonsei.ac.kr (J.L.)

* Correspondence: kimd@yonsei.ac.kr

† Current address: LG Innotek, Magok R&D Campus, Gangseo-gu, Seoul 07796, Republic of Korea.

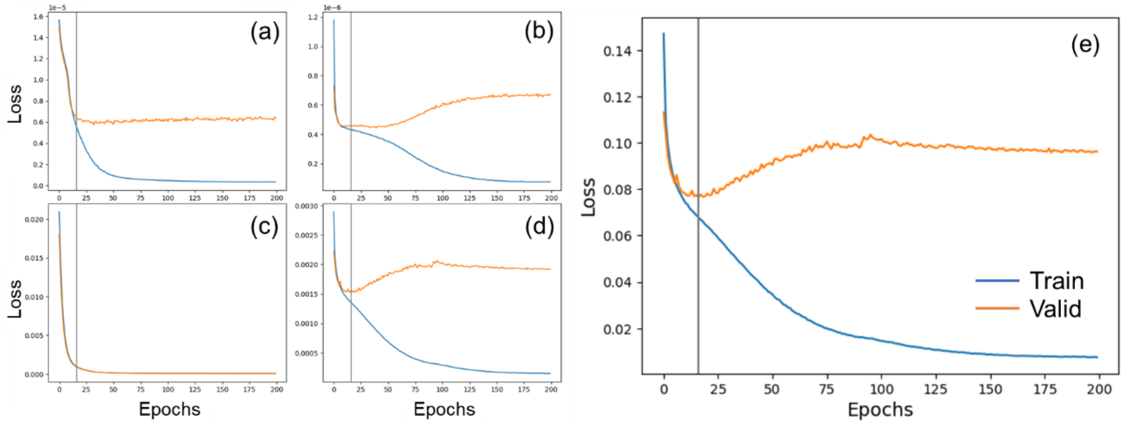

Figure S1 The loss values of the training set (blue) and the validation set (orange) during the training process of Y-Net for the four different types of losses. The losses were averaged over the entire dataset. The gray line indicates the epoch where the validation loss was minimum. (a) $\mathcal{L}_1$: scatterer phase loss, (b) $\mathcal{L}_2$: scatterer amplitude loss, (c) $\mathcal{L}_3$: scatterer location loss, (d) $\mathcal{L}_4$: scattering parameters loss, and (e) $\mathcal{L}$: final loss.

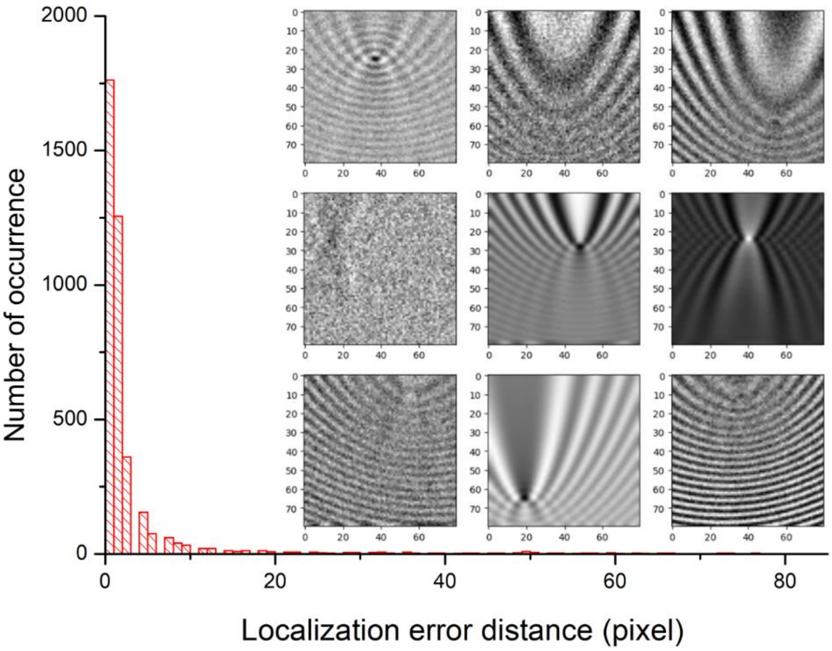

Figure S2 For quantitative analysis, we generated 4,000 images with noise added to the ground-truth model and performed prediction based on the Y-Net model. For this evaluation, we used augmentations in the Python Albumentations library to generate blur and noise. The blur was applied using a Gaussian filter with a size randomly selected between 0 and 7 pixels. The Gaussian additive noise followed a Gaussian distribution with a random variance between 0 and 0.02. The probability of applying the noise was set to 80%. Some of the scattering patterns under the noise model are provided in the inset of the figure. The result clearly demonstrates that the localization error distance was mostly within a single pixel. The number of occurrences when the localization error distance was equal to 0, 1, and 2 pixels was, respectively, 1,762, 1,255, and 360 for 4,000 total counts. With a pixel size of 93 nm, the probability of "correct" localization was approximately 84%. The noise model that we used in this simulation approximated scenarios with more significant noise compared to physical measurements. We believe that the high accuracy in such scenarios shows the promise of high localization probability in applying this method to physical images.
